# Advertisements of Specialists

**Published:** 1869-11

**Authors:** B. Joy Jeffries

**Affiliations:** Boston


Advertisements of Specialists.—*Mr. Editor:*—I have been requested to call attention to and refute the following, which appears as an editorial note in the report of the American Medical Association meeting, published in the June number of the *Richmond and Louisville Medical Journal:*

"Sichel, Donders, von Græffe, &c., placard the streets with their advertisements, and, in Edinburgh, specialists have their specialty engraved on their door-plates."

In this section of the country perhaps too many of our profession have been abroad to require me to do more than in general terms to assert that the above allegation is erroneous as regards the distinguished ophthalmic surgeons mentioned; though local authorities and custom require or permit some procedures in Europe not tolerated here.

The American Medical Association, at its late meeting, adopted the following resolution:

"*Resolved*, That private handbills, addressed to the members of the medical profession, or advertisements in newspapers or in medical journals, calling the attention of the professional brethren to themselves as specialists, be declared in violation of article one, section three, of the Code of Ethics of the American Medical Association.

The American Ophthalmological Society declares, in its Constitution, "No member of this Society shall attach to his name, in any public announcement, the title of oculist, or any similar title, or shall announce in print that he gives special or exclusive attention to special practice."

This law is strictly carried out, and two members were dismissed from our Society for violating it, at the late meeting at Newport, in July. Respectfully,

Boston, Sep. 20, 1869. B. Joy Jeffries, M. D.

